# A Systematic Review to Guide Future Efforts in the Determination of Genetic Causes of Pregnancy Loss

**DOI:** 10.3389/frph.2021.770517

**Published:** 2021-12-15

**Authors:** Andrew Z. Carey, Nathan R. Blue, Michael W. Varner, Jessica M. Page, Nathorn Chaiyakunapruk, Aaron R. Quinlan, D. Ware Branch, Robert M. Silver, Tsegaselassie Workalemahu

**Affiliations:** ^1^Department of Obstetrics & Gynecology, University of Utah Health, Salt Lake City, UT, United States; ^2^Department of Obstetrics and Gynecology, Intermountain Healthcare, Salt Lake City, UT, United States; ^3^Department of Pharmacotherapy, College of Pharmacy, University of Utah, Salt Lake City, UT, United States; ^4^School of Pharmacy, Monash University Malaysia, Subang Jaya, Malaysia; ^5^Department of Human Genetics, University of Utah, Salt Lake City, UT, United States; ^6^Utah Center for Genetic Discovery, University of Utah, Salt Lake City, UT, United States; ^7^Department of Biomedical Informatics, University of Utah, Salt Lake City, UT, United States

**Keywords:** early pregnancy loss, recurrent pregnancy loss, stillbirth, fetal death, genetics

## Abstract

**Background:** Pregnancy loss is the most common obstetric complication occurring in almost 30% of conceptions overall and in 12–14% of clinically recognized pregnancies. Pregnancy loss has strong genetic underpinnings, and despite this consensus, our understanding of its genetic causes remains limited. We conducted a systematic review of genetic factors in pregnancy loss to identify strategies to guide future research.

**Methods:** To synthesize data from population-based association studies on genetics of pregnancy loss, we searched PubMed for relevant articles published between 01/01/2000-01/01/2020. We excluded review articles, case studies, studies with limited sample sizes to detect associations (*N* < 4), descriptive studies, commentaries, and studies with non-genetic etiologies. Studies were classified based on developmental periods in gestation to synthesize data across various developmental epochs.

**Results:** Our search yielded 580 potential titles with 107 (18%) eligible after title/abstract review. Of these, 54 (50%) were selected for systematic review after full-text review. These studies examined either early pregnancy loss (*n* = 9 [17%]), pregnancy loss >20 weeks' gestation (*n* = 10 [18%]), recurrent pregnancy loss (*n* = 32 [59%]), unclassified pregnancy loss (*n* = 3 [4%]) as their primary outcomes. Multiple genetic pathways that are essential for embryonic/fetal survival as well as human development were identified.

**Conclusion:** Several genetic pathways may play a role in pregnancy loss across developmental periods in gestation. Systematic evaluation of pregnancy loss across developmental epochs, utilizing whole genome sequencing in families may further elucidate causal genetic mechanisms and identify other pathways critical for embryonic/fetal survival.

## Highlights

- The etiologies of PL and its genetic causes are poorly understood.
- Limited number of studies identified genetic pathways essential for PL.
- Genetic pathways are essential for embryonic/fetal survival and human development.
- Future research strategies require systematic evaluation of PL in families.

## Introduction

Pregnancy loss is the most common obstetric complication occurring in about 30% of conceptions (1). Approximately 10–28% of all clinically recognized pregnancies result in losses (2); of these, most occur prior to the second trimester. In the United States, losses after 20 weeks' gestation occur 1 in 160 pregnancies (3). The risk of pregnancy loss increases with a previous loss (4), suggesting that genetics may play role in families experiencing recurrent losses. Pregnancy loss recurs in about 1–2% of couples who are trying to conceive (5), and about 25% of women attempting pregnancy experience at least one loss (6). Approximately, 50% of recurrent pregnancy loss (RPL) cases are idiopathic (i.e., without any known etiologies) (7).

Genetic abnormalities (chromosomal and single-gene disorders) in the conceptus are an established etiology of pregnancy loss (8). Fetal or placental karyotype analyses allow detection of aneuploidy (chromosomal abnormalities) in 55% of first trimester losses, 35% of second trimester losses, and 7% of losses >20 weeks' gestation (9), confirming the higher rate of genetic factors contributing to losses in earlier gestation (10). However, genetic causes of losses >20 weeks' gestation may not be identified by karyotype (3). Recent studies in a large cohort of losses >20 weeks' gestation identified aneuploidy or pathogenic copy number changes as genetic causes of losses >20 weeks' gestation in 44 (9.5%) cases using chromosomal microarray analysis (3) and single-gene pathogenic variants in 13 genes (7 previously identified and 6 strong candidates) causing 15 (6.1%) losses >20 weeks' gestation using whole exome sequencing (WES) (11). Although findings from these studies may guide future research into mechanisms of pregnancy loss, they do not adequately facilitate clinical efforts to genetically screen losses across different developmental epochs (10, 12). Studies that examine DNA from products of conception, as well as the parent-offspring trio (maternal, paternal and fetal) samples, will be critical to identify causal variants and clinically significant genes. In addition, studies that identify pathways that are essential for normal and abnormal pregnancy may facilitate the discovery of novel therapeutic targets to improve pregnancy outcomes.

With the advent of next-generation sequencing (NGS), studies in the past 20 years have identified genetic pathways that are essential for *in utero* survival. In particular, some studies have shown increased likelihood of a genetic cause in early pregnancy (13), while challenges (e.g., accessibility, maternal cell contamination) remain when assessing biospecimen in products of conception from early losses. Furthermore, inconsistencies in categorizing pregnancy loss by gestational age have been noted by others (14). Using suggested standardized definitions of pregnancy loss, we underscore the importance of categorizing losses with regard to gestational age and developmental stage at the time of loss in future studies (10, 14). We conducted a systematic review to highlight genetic/multi-omic studies of pregnancy loss conducted between 2000 and 2020 and discussed key strategies to guide future relevant research efforts. Studies were classified based on developmental periods in gestation to synthesize data across various developmental epochs, allow classification by stage and etiology of loss (14) and identify common pathways (15).

## Materials and Methods

### Search Strategy

Previously published manuscripts on pregnancy loss were identified through a literature search using PubMed. The search criteria included keywords and Medical Subject Headings terms; “pregnancy loss,” “stillbirth,” “fetal death” or “fetal death,” and “placenta.”

### Study Selection

Manuscripts were eligible if they were full-text articles written in English, published between January 01, 2000 and January 01, 2020, and conducted in humans or human cell lines. We chose a search period of the last 20 years in order to identify publications that potentially utilized NGS approaches during a time when accessibility to the technologies increased. In addition, we incorporated publications investigating the placenta as there is likely a genetic contribution to placental insufficiency in some pregnancy losses (16, 17). Since the placenta is genetically similar to the fetus, it allows examination of both maternal and paternal contributions, as well as *de novo* mutations to pregnancy loss. We excluded case studies, studies with very small sample sizes (*n* < 4), descriptive articles or commentaries, infertility/non-spontaneous abortion studies and studies with non-genetic etiologies. Systematic review articles that met search criteria were further explored for relevant studies referenced therein.

### Pregnancy Outcome Classification Based on Developmental Epochs

Studies report their primary outcomes over a broad range of gestational ages [e.g., conventional definitions of stillbirth and Early Pregnancy Loss (EPL)], therefore, in the present review, we aimed to summarize studies by the pregnancy outcomes: EPL (including peri-implantational, pre-embryonic, embryonic, fetal death and early fetal death), late fetal death and losses >20 weeks' gestation, unclassified fetal death (losses assessed across gestation) and RPL. RPL is most commonly defined as ≥3 losses with ≤1 intervening live birth (5), however, we included studies that defined RPL as ≥2 losses. The recommended classifications allow evaluation across various developmental epochs, classifying losses by stage and etiology of loss (14) to help identify common pathways (15). Furthermore, the classifications may identify studies that report genetic factors with different mechanisms, e.g., genes essential for embryonic lethality and functional genes essential for human development (e.g., cardiomyopathy).

### Study Summarization

In this systematic review, we summarized the studies according to PubMed ID, first author last name and initial, year of publication, pregnancy loss outcome, predictor(s), method of assessment or study design, sample size, and tissue. We provide studies that identified candidate genes with functional pathways. For studies that did not report specific pathways, we conducted an Online Mendelian Inheritance in Man (OMIM) search to identify the roles of the reported genes in disease or functional pathways. Finally, we focused our discussion toward studies that report findings based on genetic factors that are likely causal (e.g., single-gene, autosomal and/or recessive *de novo* or inherited mutations, “intolerome,” copy number variations [CNVs], single nucleotide polymorphisms [SNPs]) (13). We summarized multi-omic studies, e.g., studies based on proteins and methylated genes that have different mechanisms than single-gene mutations or CNVs. The literature search was cross-examined by Authors. All conflicts were discussed and resolved before proceeding to systematic review.

### Systematic Review

The PRISMA 2020 checklist was utilized to ensure the manuscript conformed to the systematic review definition. Of note, this study has not been registered with a specific review protocol. There are no randomized clinical trials on genetics of pregnancy loss. Risk of bias was not assessed, principle summary measures were not utilized, and synthesis of data for a meta-analysis was not performed.

## Results

### Screened Studies Selected for Systematic Review

**Figure 1:**
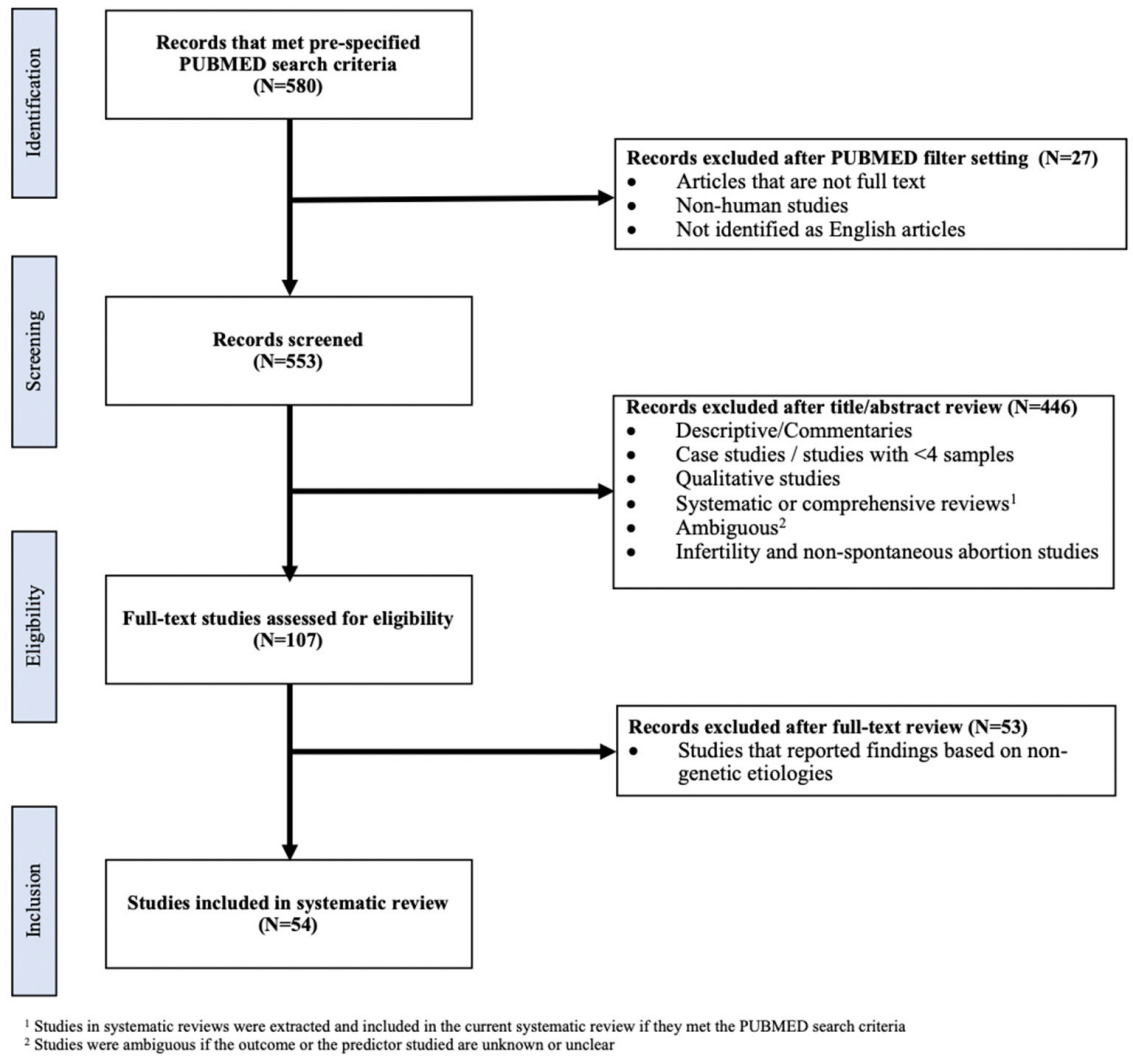
Screening process of full-text manuscripts included in systematic review.

Our search yielded 580 potential records. The PRISMA flow diagram is provided in Figure 1. After title and abstract review, 38 records were excluded after additional filters for articles that are not full text, based on non-human studies, and not identified as English articles. After title/abstract review, additional 446 records were excluded because they were either descriptive/commentaries, studies with small sample size (*n* < 4), qualitative studies, systematic or comprehensive reviews, studies based on infertility and non-spontaneous abortion, or ambiguous with critical information missing. After full-text review, 53 full-text articles that were based on non-genetic factors associated with pregnancy were excluded. In the present study, we included 54 studies that reported findings based on genetic/multi-omic etiologies involved in pregnancy loss.

### Genetic Factors Associated With EPL

Nine studies (17%) examined genetic factors in relation to EPL (Table 1). Most of the studies identified dysregulated miRNAs, epigenetic regulators which may have important role in placental development and function. The largest of these, with sample size reaching 105 participants, showed that miR-378a-3p is downregulated in early pregnancy loss (*n* = 50) compared with normal (*n* = 55) decidua (24). Hosseini et al. detected other dysregulated microRNAs (e.g., miR-135a) in maternal plasma and villous cells of women (*n* = 16) who had EPL, but the comparison group were women (*n* = 8) who underwent abortions (23). Using endocervical specimens collected prior to EPL (*n* = 20), altered protein expression patterns of extra villous trophoblast (EVT), which plays a role in proper implantation and placentation, were detected in cases compared to controls (21). The authors' ability to obtain EVT cells early from ongoing pregnancies and determine the eventual pregnancy loss occurrences may have allowed opportunities to discover novel biomarkers through global analytic approaches (21).

**Table 1:** Studies that reported genetic factors associated with EPL.

**PMID**	**First author**	**Year**	**Pregnancy loss**	**Predictor(s)**	**Method**	**Sample size**	**Tissue(s)**	**Reference**
16738225	Liu	2006	Early Pregnancy Loss	Alteration of protein expression	Proteomic analysis	12	Placental chorionic villi	(18)
23433743	Ventura	2013	Early Pregnancy Loss	Placental Expression of microRNA-17 and−19b	Matched case-control expression microRNA analysis using qPCR	31	Placental chorionic villi	(19)
24303885	Cöl-Madendag	2014	Early Pregnancy Loss	Vascular endothelial growth factor (VEGF) expression	IHC	80	Placental chorionic villi; endometrial decidua	(20)
26051097	Fritz	2015	Early Pregnancy Loss	Expression pattern of biomarker proteins in extravillous trophoblast (EVT) cells	Case-control study of trophoblast retrieval and isolation from the cervix from ongoing pregnancies	20	Endocervical specimens	(21)
30074219	Wu	2018	Early Pregnancy Loss	TET family, 5-hmC expression	quantitative reverse transcriptase polymerase chain reaction (qRT-PCR), western blotting and immunohistochemical (IHC) analyses	>3	Placental chorionic villi	(22)
29393376	Hosseini	2018	Early Pregnancy Loss	miRNAs (hsa-miRNA (miR)-125a-3p, hsa-miR-3663-3p, hsa-miR-423-5p and hsa-miR-575)	miRNA expression qRT-PCR analyses	24	Maternal plasma; placental chorionic villi	(23)
29165645	Hong	2018	Early Pregnancy Loss	miR-378a-3p expression	qRT-PCR, western blotting, luciferase reporter assays	105	Endometrial decidua	(24)
31203134	He	2019	Early Pregnancy Loss	Serum- and glucocorticoid-inducible kinase (SGK1) expression	Gene expression case-control analysis	67	Placental chorionic villi	(16)
19389728	Sarno	2009	Early Pregnancy Loss	HOX gene expression	qRT-PCR and western blotting analyses	46	Endometrial decidua	(25)

### Genetic Factors Associated With Losses >20 Weeks' Gestation

Since a standardized definition of stillbirth has not been agreed upon, studies examining loss >20 weeks' gestation were lumped together and classified based on their specific cutoffs. One study examined self-reported miscarriage or stillbirth as the primary outcome over a broad range of gestational ages and 10 studies (18%) examined losses >20 weeks' gestation as the primary outcome (Table 2). Of these 10 studies, cutoffs of 20, 22, 23, 24, and 32 weeks were utilized (Table 2). Seven studies examined the associations of genes involved in maternal thrombophilia with losses >20 weeks' gestation. The largest of these, with sample size reaching 1,830 participants, performed a candidate gene analysis (30). The only positive association was with maternal homozygous SNP in *FVL* (Factor V Leiden) gene (2/488 [0.4%] vs. 1/1380 [0.0046%]; OR = 87.4; 95% confidence interval [95%CI]: 7.9–970.9). The investigators concluded that these heritable thrombophilia genetic markers were not associated with losses >20 weeks' gestation. In another candidate gene study, pregnancy loss >22 weeks' gestation was associated with carriers (*n* = 96) of allele A of rs1800783 *eNOS* (endothelial nitric oxide synthase 3) gene in placental tissue. The *eNOS* gene may be critical for pathways involved in placental growth (28). Furthermore, a genome-wide analysis using high-resolution Illumina SNP arrays identified 24 putative novel CNVs in placental and fetal samples (*n* = 54) (27). Using a larger study with similar methodology, Reddy et al. detected normal, abnormal (pathogenic), and variants of unknown significance CNVs in 396 (74.4%) samples from pregnancy loss >20 weeks' gestation (including samples with anomalies) (3). The remainder of the studies examining losses >20 weeks' gestation utilized other techniques such as quantitative reverse transcriptase polymerase chain reaction (qRT-PCR), immunohistochemistry, and Western blot.

**Table 2:** Studies that reported genetic factors associated with losses >20 weeks' gestation.

**PMID**	**First author**	**Year**	**Pregnancy loss**	**Predictor(s)**	**Method**	**Sample size**	**Tissue(s)**	**Reference**
15963226	Wicherek	2005	Loss ≥24 weeks' gestation	Placental RCAS1 expression	Western blot method with the use of monoclonal anti-RCAS1 antibody	67	Placental	(26)
21732394	Harris	2011	Loss >22 weeks' gestation	Genomic structural variations; CNVs	Genome-wide analysis using high-resolution Illumina SNP arrays (Human CNV370-Duo)	54	Placental tissue; fetal tissue	(27)
23021696	Ferrari	2012	Loss >22 weeks' gestation	SNPs in endothelial nitric oxide synthase (eNOS) gene	Case-control candidate SNP association	96	Placental tissue	(28)
23215556	Reddy	2012	Loss ≥20 weeks' gestation	CNVs of at least 500 kb	Chromosomal microarray analysis (case-only)	532	Placental tissue; fetal tissue	(3)
26094028	Ernst	2015	Loss ≥23 weeks' gestation	Fetal copy-number variation (CNV)	Retrospective case-control microarray and qPCR analyses	94	Umbilical cord	(29)
27131585	Silver	2016	Loss ≥20 weeks' gestation	Maternal factor V Leiden; fetal PAI-1 4G/4G polymorphism	Case-control candidate single nucleotide polymorphism (SNP) association	1,830	Maternal serum; fetal cord blood; placental chorionic villi	(30)
26827667	Romagnuolo	2016	Loss >24 weeks' gestation	Lp(a) levels measurement	Retrospective observational study	630	Maternal blood leukocytes; maternal blood	(31)
26004986	Ferrari	2016	Loss >22 weeks' gestation	Placental telomere shortening	qPCR of 42 unexplained stillbirths (>22 weeks), 43 term and 15 preterm live births	100	Placental tissue	
28645573	Maiti	2017	Loss ≥32 weeks' gestation	Aldehyde oxidase 1 and G-protein-coupled estrogen receptor 1	IHC and gene expression analyses using qRT-PCR	4	Placental chorionic villi	(32)
28990860	Campbell	2018	Loss ≥24 weeks' gestation	Genetic test results, placental pathology	Review of pathology reports and collected demographic data on cases	131	Placental	(33)

### Genetic Factors Associated With RPL

Thirty-two studies (59%) examined RPL, including pre-embryonic, embryonic, and fetal losses, as the primary outcomes. There was variation in the definition of RPL across studies, with some using a minimum of two losses (34, 35) and others using a minimum of three (17, 36–42) (Table 3). The majority of RPL studies were hypothesis-based, i.e., conducted a candidate gene approach to examine SNPs in selected genes, *a priori*, and with plausible pathophysiologic pathways. Haplotype analysis conducted by Rogenhofer et al. showed that maternal blood M2 haplotype carriers with RPL (*n* = 100) in *ANXA5*, annexin 5 gene involved in coagulation, had a 3.4-fold increased RPL risk compared to controls (*n* = 500) and a 2.1-fold increased RPL risk compared to randomly selected population controls (*n* = 533) (47). SNP-prevalence analysis conducted by Jin et al. showed RPL cases (*n* = 112) carried the rs2249825 G allele in *HMGB1* (high mobility group box 1) gene in maternal whole blood more frequently than controls (*n* = 118) (48). Seyedhassani et al. compared the frequency of mutations in *BAX* gene, a pro-apoptotic gene, among RPL women (*n* = 67) and controls (*n* = 70) and showed associations between A(-179)G mutation in the *BAX* promoter and RPL (41). Quintero-Ronderos et al. sequenced the complete coding region of *THBD*, the endothelial cell receptor for thrombin gene, in women affected by RPL (*n* = 262) and showed *THBD*-p.Trp153Gly mutation might be related to RPL (54). Lastly, Masini et al. analyzed the genotype and allele frequencies of thrombin-activatable fibrinolysis inhibitor (*TAFI*) SNPs among women with (*n* = 86) and without (*n* = 72) RPL. Genotype and allele frequencies of *TAFI* +505 and +1583 SNPs were significantly different in women with RPL compared to controls (38).

**Table 3:** Studies that reported genetic factors associated with RPL.

**PMID**	**First author**	**Year**	**Pregnancy loss**	**Predictor(s)**	**Method**	**Sample size**	**Tissue(s)**	**Reference**
31396989	Zhang	2019	Recurrent Pregnancy Loss	NOD1 gene expression	Gene expression case-control analysis	38	Endometrial decidua	(43)
17099210	Kaare	2007	Recurrent Pregnancy Loss	Variations in the thrombomodulin and endothelial protein C receptor genes	Case-control family (couples) mutation detection using liquid chromatography	277	Maternal blood; paternal blood	(44)
21160146	Ticconi	2009	Recurrent Pregnancy Loss	Genotype allele frequency of Beta-Fibrinogen G-455A	Case-control study	176	Maternal blood	(45)
11857060	Wang	2002	Recurrent Pregnancy Loss	Polymorphism of the IL-1beta gene (IL1B)	Retrospective case-control study SNP frequency	59	Peripheral blood mononuclear cells (PBMCs) derived from trophoblast cell line	(36)
12874795	Choi	2003	Recurrent Pregnancy Loss	Expression of Angiogenesis and Aptosis related genes	qRT-PCR analysis	12	Placental chorionic villi	(42)
16253969	Wang	2006	Recurrent Pregnancy Loss	Maternal CD46H*2 and IL1B-511*1 Homozygosity in T Helper 1-type Immunity to Trophoblast Antigens	Case-control study	203	Trophoblast tissue	(37)
18774564	Masini	2009	Recurrent Pregnancy Loss	Thrombin-activatable fibrinolysis inhibitor (TAFI) single nucleotide polymorphisms (SNPs)	Case-control study	158	Maternal blood	(38)
21996032	Park	2011	Recurrent Pregnancy Loss	Kisspeptin expression	IHC, flow cytometry and gene expression analyses	52	Endometrial decidua; trophoblast tissue; maternal blood	(34)
20977975	Eller	2011	Recurrent Pregnancy Loss	Vascular Endothelial Growth factor-A Gene Polymorphisms	Case-control study allele frequency analysis	280	Placental tissue	(39)
20962020	Uusküla	2011	Recurrent Pregnancy Loss	Methylation Allelic Polymorphism (MAP) in Chorionic Gonadotropin beta5 (CGB5)	methylation analysis	32	Trophoblast tissue	(40)
22291743	Seyedhassani	2011	Recurrent Pregnancy Loss	Alterations of the Bax gene (a pro-apoptotic gene)	Case-control frequency of mutation detection using PCR	137	Maternal blood	(41)
22935024	Saunders	2012	Recurrent Pregnancy Loss	IgG(3) reactivity	Case and matched control comparison using Immunoprecipitation and Western immunoblotting analyses	28	Maternal serum	(35)
22505054	Kreig	2012	Recurrent Pregnancy Loss	Gene expression alterations	Case-control microarray; gene expression; pathway, gene ontology (GO) and qRT-PCR analyses.	16	Endometrial decidua	(17)
23850136	Nair	2013	Recurrent Pregnancy Loss	Inflammatory Proteins S100A8 and S100A9	qPCR and western blot analyses to examine differential expression between cases and controls	65	Endometrial decidua	(46)
23498654	Rogenhofer	2013	Recurrent Pregnancy Loss	M2 haplotype of ANXA5 gene	Comparing M2/ANXA5 genotype among 100 PCOS, 500 fertile and 533 random population control women	1,133	Maternal blood	(47)
25956264	Jin	2015	Recurrent Pregnancy Loss	HMGB1 rs2249825C/G and rs1412125T/C polymorphisms	Case-control study of PCR-restriction fragment length polymorphism assay analyses	230	Placental chorionic villi	(48)
25925347	Perfetto	2015	Recurrent pregnancy Loss	IL-22 levels	qPCR, Western blot, and IHC	20	Endometrial decidua	(49)
27535546	He	2016	Recurrent Pregnancy Loss	Early pregnancy Cx43 and VEGF mRNA and protein expression	IHC, western blot, and qRT-PCR analyses	56	Placental chorionic villi; endometrial decidua	(50)
27477959	Yan	2016	Recurrent Pregnancy Loss	1st trimester Vitamin D receptor (VDR) expression	Evaluation by IHC, confocal laser scanning microscopy (CLSM), western blot, qPCR, and enzyme-linked immunosorbent assay	80	Placental chorionic villi; endometrial decidua	(51)
27929073	Sober	2016	Recurrent Pregnancy Loss	Gene expression alterations	Case-control RNA differential sequencing (DESeq) analysis	10	Placental chorionic villi	(52)
26826164	Qiao	2016	Recurrent Pregnancy Loss	DNA alterations within exons	Case-only family WES	4	Maternal blood; paternal blood; placental chorionic villi	(53)
28345611	Kasak	2017	Recurrent Pregnancy Loss	NUP98 (embryonic stem cell development) and MTRR (folate metabolism) genes	Copy number variant (CNV) analysis of idiopathic RPL trios (mother-father-placenta) and duos (mother-placenta)	79	Maternal blood; paternal blood; placental chorionic villi	(7)
29195508	Quintero-Ronderos	2017	Recurrent Pregnancy Loss	Endothelial cell receptor for thrombin gene (THBD)	Case-control coding sequence mutation detection using bioinformatics	262	Maternal blood	(54)
29016666	Quintero-Ronderos	2017	Recurrent Pregnancy Loss	DNA alterations within exons	Case-only whole exome sequencing (WES)	49	Maternal blood leukocytes	(55)
28833278	Shehab	2017	Recurrent Pregnancy Loss	FOXP3 gene frameshift mutations (p.D303fs*87)	Whole genome sequencing of families	7	Maternal blood; unaffected offspring blood; fetal tissue	(56)
30348621	Li	2018	Recurrent Pregnancy Loss	ITI-H4 and plasma kallikrein (KLKB1)	Gene expression case-control analysis	90	Maternal serum; maternal blood	(57)
30100398	Yu	2018	Recurrent Pregnancy Loss	CREB5 expression	Genome-wide DNA methylation and gene expression analyses	100	Endometrial decidua	(58)
24557735	Papamitsou	2014	Recurrent Pregnancy Loss	Expressions of HLAG (Human Leukocyte Antigen G), CD68 (Cluster of Differentiation 68), CD56, CD16 and CD25 during pregnancy	IHC	50	Endometrial decidua	(59)
11279300	Pfeiffer	2001	Recurrent Pregnancy Loss	Human leukocyte antigen (HLA)-G genotype	Case-control comparison of haplotypes	130	Maternal blood; paternal blood	(60)
16403802	Kaare	2006	Recurrent Pregnancy Loss	Homozygous mutations in the Amnionless (AMN) gene	Case-only Families (couples) sequence variation detection using liquid chromatography	85	Maternal blood; paternal blood	(61)
25457193	Agrawal	2015	Recurrent Pregnancy Loss	HLA-G 5′ upstream regulatory region SNPs	Case-control comparison of haplotypes	200	Maternal blood; paternal blood	(62)
24621454	Gharesi-Fard	2014	Recurrent Pregnancy Loss	Proteins involved in proliferation and migration of endothelial cells as well as control of coagulation	Differential expression analysis using qPCR and Western blot techniques	10	Placental tissue	(63)

Genome-wide association studies of RPL were also conducted to highlight genetic variants with relevant functional pathways. For example, Kasak et al. examined placental and parental genome-wide CNV profiles of idiopathic RPL trios (*n* = 25 parental blood, *n* = 13 placental) and duos (*n* = 8 maternal blood, n = 9 placental), and detected CNVs in *NUP98* and *MTRR* genes (7). *NUP98* (Nucleoporin 98 And 96 Precursor) and *MTRR* (5-Methyltetrahydrofolate-Homocysteine Methyltransferase Reductase) genes are implicated in embryonic stem cell development and folate metabolism, respectively (7). Another genome-wide association study was reported by Yu et al. (31) but the study identified DNA methylation and gene expression, mechanisms that are also modulated by environmental factors (31). The study suggested hypo-methylation in *CREB5* gene in the decidual tissue was associated with RPL (58).

Next generation sequencing approaches further identified deleterious mutations that are likely causal. For example, by conducting whole exome sequencing (WES) using parental blood and placental chorionic villi samples, Qiao et al. (53) detected compound heterozygous deleterious mutations affecting *DYNC2H1* and *ALOX15* genes, both critical for early development, in two out of four families with RPL. Among unrelated women (*n* = 49) affected by RPL, Quintero-Ronderos et al. conducted WES in maternal leukocytes and detected 27 coding variants in 22 genes among 41% of the women. The affected genes, which were enriched by potentially deleterious sequence variants, belonged to distinct molecular cascades playing key roles in implantation (55). Furthermore, Shehab et al. conducted WGS analyses using maternal blood, unaffected offspring blood and fetal tissue in families (*n* = 7) with recurrent fetal death and detected a frameshift mutation in *FOXP3* gene. The authors confirmed the mutation in the affected fetal tissue using Sanger sequencing.

### Genetic Factors Associated With Unclassified Pregnancy Loss

Three studies were based on unclassified pregnancy loss, assessed over a broad range of gestational ages (Table 4). Cochery-Nouvellon et al. conducted a candidate gene study using 3,218 case (experienced embryonic loss at <10 weeks and fetal loss ≥10 weeks gestation) and 6,436 control mother-father pairs, the largest 1:2 matched case-control family-based study included in our review (66). The authors reported that the A6936G allele of *PROCR*, an endothelial protein C receptor gene involved in coagulation (Table 5), in maternal and paternal blood is associated with fetal death. The authors confirmed the association between candidate gene Factor V Leiden (*F5*), also involved in coagulation, and fetal loss, but pointed out that relationship between thrombophilias and pregnancy loss varies according to ethnicity and loss type. Alonso et al. (64) also examined mutations in the *F5* gene in first-trimester abortions (at ≤ 12 weeks of gestation), second-trimester abortions (at 13–22 weeks of gestation), and fetal death (at ≥23 weeks) of mothers (*n* = 75). The presence of thrombophilia in 75% of the women combined with a mutation in *F5* gene was marginally associated with intrauterine fetal death (*P* = 0.04; OR = 12; 95%CI: 1.44–102).

**Table 4:** Studies that reported genetic factors associated with unclassified pregnancy loss.

**PMID**	**First author**	**Year**	**Pregnancy loss**	**Predictor(s)**	**Method**	**Sample size**	**Tissue(s)**	**Reference**
12439528	Alonso	2002	Unclassified Pregnancy Loss	Mutations of factor V Leiden, methylenetetrahydrofolate reductase, and prothrombin gene	Case-control ELISA analysis	150	Maternal blood	(64)
30136429	Mehandjiev	2018	Unclassified Pregnancy Loss	MTHFR C677T TT genotype and T allele	Cross-sectional study	243	Endometrial decidua	(65)
19806250	Cochery-Nouvellon	2009	Unclassified Pregnancy Loss	A6936G allele of the endothelial protein C receptor (EPCR) gene (PROCR)	1:2 case-control study	9,654	Maternal blood; paternal blood	(66)

**Table 5:** Reported genetic/multi-omic pathways in relation to gestational age specific pregnancy.

**Pregnancy loss phenotype**	**Genes, microRNAs, mRNAs, or chromosomes**	**Functional pathway**	**Number of studies**
Early pregnancy loss			7
	SGK1, miR-575, miRNA-17, miRNA-19b, VEGF	Placental function	
	TET family, 5-hmC	Epigenetic reprogramming	
	miR-125a, miR-3663-3p	Mitosis, meiosis, cell cycle progression	
	miR-3663-3p, miR-135a, miR-122, let-7, miR-378a-3p	Apoptosis	
	miR-125a	Hematopoiesis	
	miR-125a, miR-135a	Implantation	
	HOX family	Endometrial function	
Losses ≥20 weeks' gestation			6
	F5, PAI-1, eNOS	Coagulation	
	AOX-1, GPER	Oxidation and cellular aging	
	LPA	Lipoprotein synthesis	
	Ch 1q31.3, NOS3, RCAS1	Inflammation and immunity	
	eNOS	Mitosis, meiosis, cell cycle progression	
	eNOS	Vascular tone	
Recurrent pregnancy loss			32
	NOD1, ITI-H4, KLKB1, IL-22, HLAG, CD16, CD68, CD56, S100A8, S100A9, KISS1, IL1B, CD46, FOXP3, NLRP2, NLRP5, NLRP7, IDO2	Inflammation and immunity	
	CREB5, DYNC2H1, PLCD4, OSBPL5, STIL	Mitosis, meiosis, cell cycle progression	
	CREB5, BAX, CASP9	Apoptosis	
	NUP98, IFT122, APAF1, CASP9, CSPP1, NLRP5, PADI6	Embryonic development	
	MTRR, VDR	Folate and other vitamin metabolism	
	Cx43, VEGF, ALOX15	Placental function	
	Cx43, VEGF, VEGFA, FLT1, EPAS1	Angiogenesis	
	ANXA5, TAFI, THBD, FGA, FGB, PROCR	Coagulation	
	KISS1, CHRNA1, RYR1, MUSK	Cell signaling	
	CGB5	Implantation	
	KIF14, IFT122, DYNC2H1	Ciliogenesis	
	MMP10	Extracellular matrix organization	
	CAPS	Ion transport	
Unclassified fetal death			3
	PROCR, F5, F2	Coagulation	
	MTHFR	Folate and other vitamin metabolism	

### Genetic/Multi-Omic Pathways of Pregnancy Loss

**Figure 2:**
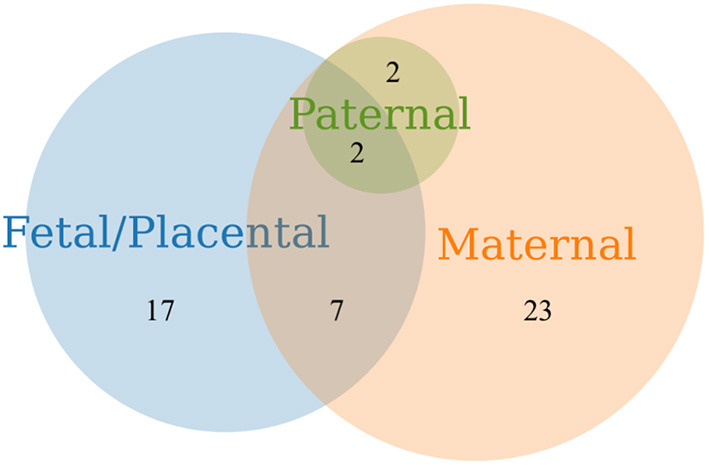
Venn diagram of pregnancy loss studies that examined genetic factors assessed in products of conception obtained from fetal/placental and parental samples.

Among the 54 studies included in this review, 26 (48%) examined placental tissue (e.g., chorionic villous tissue and trophoblast cells) and reported placental genetic factors associated with pregnancy loss across the developmental epochs. Two studies (4%) incorporated samples from parent-offspring trios (maternal, paternal and fetal/placental) and identified genetic factors related to recurrent losses. Twenty-three studies (53%) examined genetic factors assessed in the maternal tissue samples only (Figure 2). Multiple genetic pathways associated with embryonic and fetal survival may play a role in pregnancy loss. The reported pathways are essential for placental function, epigenetic reprogramming, embryonic development and several critical cellular functions (Table 5).

## Discussion

In this review we identified 54 research studies that reported genetic/multi-omic etiologies underlying pregnancy loss. Twenty-six studies examined DNA from placental and/or fetal tissues, including two studies with maternal and paternal samples, and supported their findings on genetic abnormalities associated with pregnancy loss. Based on data from studies included in this review, multiple genes with functional pathways that may be essential for embryonic/fetal survival were discussed.

### Genetic Factors Associated With Pregnancy Loss

Eight studies reported genetic/multi-omic etiologies of EPL, however, the studies examined miRNAs, including other epigenetic regulators and proteins that require utilization of expensive targeted assays (e.g., qRT-PCR and immunohistochemistry). Epigenetic mechanisms may play an important role in placental development and function, but are also modulated by environmental factors (7). Indeed, the etiology of many pregnancy losses could be multifactorial, including genetic and environmental factors; however, in some couples, pregnancy loss can be inherited as a Mendelian trait (i.e., monogenic form) (67). Despite the strong genetic underpinnings underlying EPL (10, 68), evidence for causal genetic variants is lacking.

Among genome-wide association studies of pregnancy loss at 20 weeks' gestation or more, two studies utilized chromosomal microarray, a higher resolution and enhanced sensitivity method that allowed unbiased detection of pathogenic abnormalities (3, 27). These studies by Reddy *et* al and Harris *et* al detected 24 putative novel CNVs in 54 placental and fetal samples from losses >20 and 22 weeks' gestation, respectively, and genetic abnormalities explained 41.9% of idiopathic cases (3, 27). A recent study, that was not included in our review due to its publication date, improved these findings by utilizing NGS approach that allowed detection of the *de novo* lethal mutations and the “intolerome” (i.e., genes that are critical for human development, the loss of which is incompatible with life) (11). Using the maternal and fetal samples, enrichment of loss-of-function variants in genes that are intolerant to variation in the human population were observed. This suggested dramatic and progressive increases in the proportion of losses >20 weeks' gestation with likely causative genetic abnormalities, however, the genetic etiologies of 40% of idiopathic cases remain to be elucidated. Due to unavailability of paternal samples in the previous studies, they could not detect compound heterozygous variants, distinguish pathogenic *de novo* from inherited variants and consequently could not explain significant proportion of idiopathic cases. Additional efforts were made by Cochery-Nouvellon et al. (66) that utilized mother-father duos with larger sample size. However, the study was a candidate gene study and showed limited evidence of association between coagulation pathway genes and unclassified pregnancy loss.

Among thirty-two studies that reported genetic etiologies of RPL, making up the majority of studies included in this review, two utilized an NGS approach in families to identify deleterious mutations that are likely causal (53, 56). Using WES analysis in parental blood and placental chorionic villi samples, Qiao et al. (53) detected compound heterozygous deleterious mutations affecting *DYNC2H1* and *ALOX15*, genes critical for early development, in two out of four families with RPL. By conducting WGS followed by Sanger sequencing validation analyses, Shehab et al. (56) detected frameshift mutation in *FOXP3* gene that is critical for the function of regulatory T cells in families affected by recurrent intrauterine fetal death. Other genes such as loss-of-function risk variants and inherited pathogenic mutations in intolerant genes were not identified, potentially due to the lack of larger parent-offspring trio studies.

### Guide to Next Steps in Determining Genetic Factors Associated With Pregnancy Loss

While chromosomal microarray, the current clinical guideline for genetic evaluation of losses >20 weeks' gestation, enhanced the ability to detect microdeletions and duplications beyond the resolution of standard karyotype, additional detailed diagnostic yields will require utilization of NGS approach. Efforts are underway to apply this technology to losses >20 weeks' gestation (69).

With the advent of NGS, monogenic disorders (including *de novo*, inherited autosomal-dominant/autosomal-recessive mutations, and SNPs) that are either lethal, known to cause disease, or dramatically increase risk of pregnancy loss in families can be identified. *De novo* mutations occur as likely penetrant variation in a Mendelian gene and could explain sporadic cases of pregnancy loss. Point mutations, other genetic variations such as CNVs (genomic deletions or duplications), as identified by studies in this review, may also occur *de novo*. The added contribution of novel *de novo* missense variants to losses >20 weeks' gestation was estimated by pulling all rare and damaging novel missense variants in the study (11). Therefore, the authors estimated a bound on the diagnostic yield in known genes associated with losses >20 weeks' gestation between the previously reported yield (4.5%) vs. the present yield (13.4%; 36/268 cases). However, without parental genotype information, the study remained at the lower bound of the diagnostic yield. Consistent with other diagnostic studies, the diagnostic yield using parent-offspring trios is estimated to be up to three-fold higher compared with studies that use singletons (70).

Combined with identification of *de novo* mutations, other single gene abnormalities may be used to provide prognosis based on data from other patients with similar mutations (71). Such monogenic forms may be associated with extreme phenotypes and early losses, but this is not always the case. Studies that show familial aggregation of pregnancy loss may help clarify whether losses that occur early in gestation and a positive family history exists, suggesting autosomal-dominant transmission of risk alleles. To prove whether the mutations appeared in the germline of the probands as *de novo* mutations, parental DNA assessment is required (67).

Challenges still remain in clinical applications of genome sequencing and validating the results from sequencing using maternal cell-free DNA, chorionic villus sampling and amniocentesis. Suggested strategies to overcome these challenges include serial assessment of genotypes, phenotypes and ‘omics data over the course of the pregnancy (e.g., genomics, transcriptomics, metabolomics) (10, 68). Molecular diagnostic evaluations rely on databases (e.g., OMIM) and guidelines of the American College of Medical Genetics and Genomics with characteristics designed to enrich for pathogenicity in Mendelian disease genes (11). In these databases, lethal phenotypes are especially poorly represented. Other strategies for gene discovery, including determination of the “intolerome” are likely to reveal new genotype-phenotype correlations and shed light on the human “intolerome,” conditions incompatible with life resulting in fetal demise (11, 13). Studies that incorporate DNA sequencing in affected and unaffected families, designed as case-control trio studies, will help in determination of the “intolerome” by identifying novel embryonic-lethal or fetal-lethal variants that are not seen in unaffected families. Using WGS in parent-offspring trios, 60–80 high confidence *de novo* mutations per individual can be identified (67). Compared with WES, WGS may further expand the spectrum of causal *de novo* mutations by allowing for a better coverage of the exome and identification of non-coding variants.

### Limitations and Strengths of the Systematic Review

Although PubMed search is a comprehensive retrieval tool appropriate for systematic review of journal research in health care, other search methods (e.g., Embase, Web of Science) were not utilized. Restricted MeSH terms applied in PubMed may have excluded other studies pertinent to the present systematic review. To provide a more comprehensive review of the literature, we reviewed and included studies within review articles that matched eligibility in our search criteria. In addition, we independently explored OMIM to report and confirm genetic pathways and functional effects of the reported genes.

### Guide to Next Steps

Experts have recommended categorization of pregnancy loss as: <10 weeks gestational age (termed early pregnancy loss), 10–19 weeks and 6 days of gestation (termed fetal death), and 20 or more weeks gestation (termed stillbirth). EPL was further subdivided into peri-implantational loss before 5 weeks, pre-embryonic loss from 5 to 5 weeks and 6 days of gestation, and embryonic loss from 6 to 9 weeks and 6 days of gestation (14). Similarly, fetal death can be subdivided into early fetal death, defined as losses between 10 and 15 weeks and 6 days of gestation, and late fetal death, losses from 16 to 19 weeks and 6 days of gestation (14). These classifications may identify studies that report genetic factors with different mechanisms, e.g., genes essential for embryonic lethality and functional genes essential for human development (e.g., cardiomyopathy). Additionally, assessment of losses at different stages of pregnancy may help identify pathways essential for *in utero* survival at critical stages of development.

## Conclusion

Pregnancy loss is multi-factorial, but recent studies identified genetic pathways essential for embryonic and fetal survival. Further research systematically evaluating pregnancy loss across various developmental epochs and utilizing NGS in families may identify single-gene mutations causing embryonic/fetal loss and that are not found in healthy controls. Identification of such genes and their pathways may provide novel biomarkers for risk stratification and therapeutic targets to improve pregnancy outcomes.

## Data Availability Statement

The original contributions presented in the study are included in the article/supplementary material, further inquiries can be directed to the corresponding author.

## Author Contributions

The literature search was conducted and cross-examined by AC and TW. AC, NB, MV, TW, and RS directed its implementation. AC and TW drafted the manuscript. All authors reviewed the article and revised it critically for important intellectual content, and all authors provided final approval of the draft being submitted.

## Funding

Research reported in this publication was supported in part by the National Center for Advancing Translational Sciences of the National Institutes of Health under Award Number 1UL01TR002538. This funding as well as funding from the University of Utah support open access publication fees.

## Author Disclaimer

The content is solely the responsibility of the authors and does not necessarily represent the official views of the National Institutes of Health.

## Conflict of Interest

The authors declare that the research was conducted in the absence of any commercial or financial relationships that could be construed as a potential conflict of interest.

## Publisher's Note

All claims expressed in this article are solely those of the authors and do not necessarily represent those of their affiliated organizations, or those of the publisher, the editors and the reviewers. Any product that may be evaluated in this article, or claim that may be made by its manufacturer, is not guaranteed or endorsed by the publisher.
